# Evaluation of an herbal therapy to alleviate acute pain and stress of disbudded dairy calves under organic management^1^

**DOI:** 10.1093/tas/txab044

**Published:** 2021-03-07

**Authors:** Hannah N Phillips, Bradley J Heins

**Affiliations:** 1 Department of Animal Science, University of Minnesota, Saint Paul, MN 55108, USA; 2 West Central Research and Outreach Center, University of Minnesota, Morris, MN 56267, USA

**Keywords:** behavior, calf, cortisol, disbud, herbal medicine, pain

## Abstract

The objective of this experiment was to evaluate a herbal therapy used in place of standard synthetic analgesia to mitigate disbudding pain of dairy calves. For this experiment, 54 calves were randomly assigned to one of three treatments: 1) local anesthetic lidocaine given as a cornual nerve block before cautery disbudding (AD); 2) sham disbudding (SD); or 3) herbal tincture (Dull It, Dr. Paul’s Lab, Mazomanie, WI) composed of white willow (*Salix alba* L.) bark, St. John’s wort (*Hypericum perforatum* L.), chamomile (*Matricaria recutita* L.), arnica (*Arnica montana* L.), and fennel (*Foeniculum vulgare* Mill.) administered orally before and after cautery disbudding (TD). Behaviors were assessed during disbudding, and behaviors and blood plasma cortisol concentrations were assessed following disbudding. Tail wag, head movement, forcing ahead, and kick rates recorded during disbudding were similar among treatments. When averaged across the 360-min observation period following disbudding, injury-directed behavioral rates of head jerks, head shakes, horn bud scratches, and head rubs were greater (*P* ≤ 0.03) for calves in the AD group than calves in the SD group, calves in the TD group had greater (*P* < 0.01) horn bud scratch and head rub rates compared to calves in the SD group, and calves in the AD group had a greater (*P* < 0.01) horn bud scratch rate than calves in the TD group. Calves in the AD group took 1.6 [95% confidence interval (CI) = 1.0 to 2.4, *P* = 0.03] times longer to lie down after disbudding compared to calves in the TD group. Serum cortisol concentrations were greater (*P* ≤ 0.01) for calves in the TD group compared to calves in the SD group at 10, 30, and 90 min after disbudding. At 30 min after disbudding, calves in the AD group had 5.8 ng/mL (95% CI = −1.1 to 12.7 ng/mL, *P* = 0.02) greater serum cortisol compared to calves in the SD group, while calves in the TD group had 14.3 ng/mL (95% CI = 1.5 to 27.1 ng/mL, *P* < 0.01) greater serum cortisol than calves in the AD group. In conclusion, neither the local anesthetic lidocaine nor the orally administered herbal tincture attenuated both acute injury-directed behaviors and blood plasma cortisol concentrations in disbudded calves, and the tincture was clearly less effective at mitigating cortisol; therefore, additional analgesic may be required to properly manage disbudding pain effectively.

## INTRODUCTION

Cautery horn bud removal (i.e., disbudding) of young calves is a common yet painful procedure practiced on dairy farms. Pain inflicted during the cautery disbudding procedure has been previously verified by using quantitative behavioral measurements, including rates of head movements, tail wags, and vocalizations (Graf and Senn, 1999; Grøndahl-Nielsen et al., 1999; Doherty et al., 2007). Acute pain following disbudding has been documented in numerous previous studies by evaluating blood plasma/serum cortisol concentrations, and behaviors focused around the horn bud wounds, such as ear flicks, head rubs, and head shakes (Graf and Senn, 1999; Grøndahl-Nielsen et al., 1999; Faulkner and Weary, 2000; Heinrich et al., 2009; Stilwell et al., 2012; Huber et al., 2013; Stock et al., 2016). Pain following disbudding has also been previously assessed by evaluating a range of behaviors, including lying/standing, maintenance behaviors, and rumination (Grøndahl-Nielsen et al., 1999; Faulkner and Weary, 2000; Doherty et al., 2007; Stilwell et al., 2012).

Organic dairy producers have limited analgesic options for mitigating pain in dairy calves undergoing cautery disbudding. In the United States, the use of synthetic therapies for mitigating disbudding pain in organic dairy calves is restricted by regulations set forth by the U.S. Department of Agriculture (USDA) National Organic Program (NOP), which maintains official federal standards for organic production practices (USDA-AMS-NOP, 2020). Lidocaine is a commonly used synthetic substance that is approved for use in organic-certified calves and alleviates disbudding pain by providing local analgesia. Lidocaine induces a localized insensitivity in the horn bud area within 2–5 min and has a functional duration of approximately 90 min (Coetzee, 2013). Previous studies agree that lidocaine is effective at reducing escape and struggle behaviors during disbudding, acute injury-directed behaviors up to 2 h after disbudding, and acute blood plasma/serum cortisol concentrations up to 1.5–3 h after disbudding (Graf and Senn, 1999; Grøndahl-Nielsen et al., 1999; Doherty et al., 2007). However, the injection and restraint required for administering lidocaine potentially may cause pain and stress for calves (Jimenez et al., 2019), and the use of lidocaine prior to disbudding may prolong pain following the procedure (Graf and Senn, 1999; Stilwell et al., 2012). As a possible response to these shortfalls, an emerging interest in nonsynthetic alternatives for reducing disbudding pain in organic calves currently exists. In general, organic producers are familiar with using naturally derived therapies, such as herbal-based products for the treatment of mastitis in dairy cows (Pol and Ruegg, 2007). A survey of over 189 organic dairy farms in the United States reported that 21% used a naturally derived therapy as pain management for horn removal procedures as opposed to synthetic therapies (Bergman et al., 2014). Naturally derived products—which must first be approved by the farm’s NOP accredited agency—may represent potential analgesic options for mitigating cautery disbudding pain in organic dairy calves, but this hypothesis must first be evaluated under experimental conditions.

Research on the efficacy of alternative therapies used in organic livestock production is needed to verify that their use indeed improves animal welfare. Disbudding represents a major animal welfare concern among industry and nonindustry stakeholders due to the pain the procedure inflicts (Robbins et al., 2015; Ventura et al., 2015). Previous surveys of over 290 organic dairy producers and veterinarians in the United States recognized that the deficit in knowledge about effective organic-approved practices jeopardizes animal welfare (O’Neill and Wells, 2013; Pereira et al., 2013). Thus, the use of ineffective alternative practices represents a major threat to organic dairy animal welfare. In a review of dairy industry changes that affect animal welfare, Barkema et al. (2015) proposed that future research should focus on identifying effective organic-approved alternative remedies. The hypothesis of this experiment was that calves receiving a local anesthetic before disbudding, an herbal tincture before disbudding, or sham disbudding with no treatment would differ in their pain responses during and after hot-iron disbudding. Therefore, the objective of this experiment was to evaluate pain-associated behaviors and cortisol concentration of dairy calves that received either an experimental herbal tincture prior to cautery disbudding, the current standard local anesthetic procedures prior to cautery disbudding, or no treatment prior to sham disbudding.

## MATERIALS AND METHODS

### Animal Housing and Care

The University of Minnesota Institutional Animal Care and Use Committee approved all animal care and procedures specific to this experiment (protocol number 1508-32864A). This experiment was conducted at the University of Minnesota West Central Research and Outreach Center in Morris, MN, from May to July 2016 using 54 preweaned female calves aged from 35 to 57 d (mean ± standard deviation = 44 ± 1 d). This age range represented the approximate national average for age at disbudding on dairy operations in the United States (USDA, 2018). Calves used in this experiment were either pure Holstein or a crossbreed as described by Heins et al. (2010). Calves were housed in groups of 10 in straw-bedded pens consisting of a three-sided shelter (3.7 × 6.1 m) with an equal-sized outdoor area. Calves were fed once daily in quantities of 6 L per calf of unprocessed organic whole milk at 0800 h as described by Kienitz et al. (2017).

Beginning 10 d prior to the experiment, calves were acclimated to halter restraint and human handling by increasing their exposure to experimental conditions incrementally each day from 30 min on the first day to 8 h on the last day. During the acclimation period, handlers would periodically visit calves to touch their horn buds and neck. The pens were scheduled for disbudding on separate days when the youngest calf in the pen reached 5 wk of age and when precipitation was not anticipated. After calves were offered milk on the days of the acclimation period and on the day of the experiment, calves were secured to the perimeter fence of the outdoor portion of the pen using a halter and lead. Each calf had enough lead (0.9 m) to lie down, stand up, drink ab libitum water from a 3.8-L bucket fastened to the fence, and interact with adjacent calves that were 1.5 m apart.

### Catheter Placement

Catheters were placed into the jugular vein of calves 24 h prior to disbudding. While calves were restrained in a chute equipped with a head lock (Caf-Cart, Raytec, Ephrata, PA), hair was clipped around the horn bud area and in a 12-cm band around the neck. The area of catheter placement was surgically prepared with alternating povidone-iodine and 70% isopropyl alcohol scrubs. The hypodermis of the surrounding catheter site was anesthetized by infiltrating 2 mL of 2% lidocaine (Vedco, Saint Joseph, MO). The jugular was punctured with a 14-gauge × 133-mm peripheral venous catheter (BD Angiocath, Becton Dickinson, Franklin Lakes, NJ) and the needle was removed, so only the tube remained. Bandage tape was attached to the port and adhered to the neck using super glue (Gorilla Glue, Cincinnati, OH). An interlinking 190-mm extension set (Baxter Healthcare, Deerfield, IL) was fastened to the port and secured to calves with 76-mm wide bandage tape (Elastikon, Johnson & Johnson, New Brunswick, NJ) loosely around the neck. The catheters were flushed with 3 mL of heparin saline solution containing 130 IU of heparin per milliliter of saline and capped immediately following placement and during the evening prior to the experiment.

### Experimental Design

This experiment was performed as a generalized randomized complete block design. The sample size for this experiment was determined using methods described by Guo et al. (2013) and the GLIMMPSE software for repeated measures designs (Kreidler et al., 2013). Only expected results for sham disbudding (SD) and disbudding after a lidocaine cornual nerve block (AD) were used to calculate sample size. The expected means and standard deviations for key behaviors of head movements during disbudding and head shakes at 60, 120, 180, and 240 min after disbudding were from Graf and Senn (1999). The expected means and standard deviations for cortisol at 60, 180, and 360 min after disbudding were from Stilwell et al. (2012). The expected effect sizes between treatments for head movements during disbudding, average head shakes after disbudding, and average cortisol after disbudding were 1.1, 0.9, and 2.5, respectively. For the sample size calculations for head shakes and cortisol after disbudding, a LEAR model with a base correlation of 0.50 and decay rate of 0.30 was used in the GLIMMPSE online power and sample size software (https://glimmpse.samplesizeshop.org) to account for repeated measures. The estimated sample sizes needed to achieve a power >0.80 for head movements during disbudding, head shakes after disbudding, and cortisol after disbudding were 14, 6, and 8 calves per group, respectively. The maximum required sample size from these calculations was inflated by 30% to account for any potential dropped calves (14 × 1.30 = 18). Fifty-four calves were used for this experiment. Nine calves from each of the six pens (i.e., blocks) were randomly assigned to one of three treatments: 1) local anesthetic lidocaine given as a cornual nerve block before cautery disbudding (AD; *n* = 18); 2) sham disbudding (SD; *n* = 18); or 3) oral herbal tincture (Dull It, Dr. Paul’s Lab, Mazomanie, WI) administered before and after cautery disbudding (TD; *n* = 18). A local anesthetic was selected as a positive control treatment since this is the most widely used synthetic pain mitigation therapy used for disbudding calves on organic dairy farms, and the use of multimodal pain therapy is rarely implemented (Vasseur et al., 2010; Bergman et al., 2014). Treatments were balanced for sire breed and age (Table 1). The disbudding order within a pen was completely randomized.

**Table 1.** Distribution of calves by treatment and age, and treatment and sire breed

	Treatment^*a*^		
Item	AD	SD	TD
Sire breed, count			
Holstein	6	8	8
Jersey	3	2	3
Montbéliarde	2	2	2
Normande	2	1	1
Swedish Red	5	5	4
Day of age, mean ± SD	45 ± 6	44 ± 6	44 ± 6

^*a*^Treatments: AD = local anesthetic lidocaine 5 min prior to cautery disbudding; SD = sham disbudded; TD = oral tincture 2 min prior to and immediately after cautery disbudding.

### Treatment Administration

Ten minutes prior to disbudding, calves were restrained in a chute equipped with a head lock directly outside of the pen. Calves in the AD group received 5 mL of 2% lidocaine per side 5 min prior to disbudding. For each side, the needle (20 gauge × 19 mm) was inserted into the depression parallel to the temporal line pointed upward midway between the eye and horn bud, then 4 mL of lidocaine was administered into the cornual nerve and 1 mL was fanned around the nerve. Calves in the SD group did not receive any analgesic therapy, and disbudding was simulated by applying an unheated cautery iron (Inline Dehorner, Guilbert Express, New York, NY) to the horn buds of the restrained calf. Calves in the TD group received 2 mL of the herbal tincture sublingually 2 min prior to disbudding and 2 mL immediately after disbudding via a syringe. One person administered the lidocaine and tincture treatments throughout the experiment. Calves in a pen were cautery or sham disbudded 15 min apart and all calves in the experiment were cautery or sham disbudded between 1000 and 1440 h. Cautery disbudding was performed using a pistol grip cautery iron fueled by a butane/propane/propene mix (Express Dehorner, Guilbert Express, New York, NY). Cautery and sham disbudding were performed by one personnel who was blind to treatments for the cautery disbudded calves.

The dose and administration instructions for the tincture were in accordance with manufacturer guidelines. The tincture was previously marketed as a therapy to mitigate pain and stress related to castration and disbudding procedures for cattle, deer, goats, and sheep. It had been approved for use by many third-party organic certification agencies and had demonstrated popularity among organic dairy farmers for disbudding purposes. The tincture is comprised of (in order of greatest to least inclusion): ethanol, apple cider vinegar, white willow (*Salix alba* L.) bark, St. John’s wort (*Hypericum perforatum* L.), chamomile (*Matricaria recutita* L.), arnica (*Arnica montana* L.), and fennel (*Foeniculum vulgare* Mill.).

### Data Collection

Blood was collected at baseline (10 min prior to disbudding) and 1, 30, 90, 210, and 450 min following disbudding by discarding the first 3 mL and collecting the following 3 mL of blood, which was immediately transferred to serum separation tubes (BD Vacutainer, Becton Dickinson, Franklin Lakes, NJ) and stored at 4 °C. Tubes were centrifuged and serum was collected and maintained at −40 °C until serology. Catheter patency was maintained by flushing with 3 mL of a heparin saline solution containing 13 IU of heparin per milliliter of saline after each blood collection.

Escape and struggle behaviors during disbudding were documented from audio/video recordings of calves from five pens (45 calves). A camera (iPad 3, Apple Inc., Cupertino, CA) was placed 1 m above calves to enable a full view of each calf’s body during the disbudding procedure. Frequencies of tail wags, head movements, forces ahead, kicks, vocalizations, falls, and rears were counted for the duration of restraint from the moment the cautery iron made contact with the first horn bud to the moment the cautery iron was released from the second horn bud. The duration of cauterization was also recorded.

Behaviors during and after disbudding were documented from video recordings of calves from four pens (36 calves). Two cameras were placed on opposite sides of each pen 1.5 m above the ground. For each calf, twenty-one 5-min continuous observations were performed at baseline (60, 40, and 20 min prior to disbudding) and every 20 min following disbudding over the course of a 360-min observation period. Frequencies of ear flicks, head jerks, head shakes, head rubs, oral behaviors, horn bud scratches, and transitions, and durations of standing and ruminating were hand-recorded during each observation. An ethogram for behaviors recorded in the experiment is in Table 2. The ethological evaluation of disbudded calves was intended to assess pain since behavioral adaptations can be observed in animals subjected to pain (Morton and Griffiths, 1985). Tail wagging, head movements, forcing ahead, rapid leg movements, and vocalizations are all behavioral adaptations frequently observed in ethological evaluations of calves during the cautery disbudding procedure (Graf and Senn, 1999; Caray et al., 2015), while ear flicking, exaggerated or rapid head movements, horn bud scratching, increased transitions between standing and lying, and variations in standing/lying, ruminating, and oral manipulations are all behavioral adaptations commonly recorded in ethological evaluations of calves following cautery disbudding (Grøndahl-Nielsen et al., 1999; Heinrich et al., 2010; Stilwell et al., 2012). A single treatment-blinded observer assessed and documented behaviors. Interclass correlation coefficients of behavior observations for intrareliability were >0.90.

**Table 2.** Ethogram of behaviors assessed before, during, and after the disbudding procedure

Behavior^*a*^	Description
Observations during disbudding	
Tail wag	A rapid lateral swing of the tail from one side of the body to the other
Head movement	A distinct movement of the head away from the cautery iron or upward. Not recorded during a rear or force ahead
Force ahead	A push forcefully forward
Kick	A lift and strike with a hind leg
Vocal	An oral sound, such a bellow or bawl
Fall	A complete drop to the ground or onto knees
Rear	An attempt to lift forelegs
Observations before and after disbudding	
Injury directed	
Ear flick	A rapid movement of one or both ears. Not recorded during a head shake. Recorded as a new event once ears rested for >2 s
Head jerk	An exaggerated head movement, such as a bob, jolt, or turn. Recorded as a new event once head rested for >2 s
Head shake	A rapid head tilt from side-to-side while twisting neck. Recorded as a new event when head rested for >2 s
Head rub	A back and forth movement of the head on any object. Not recorded during a horn bud scratch. Recorded as a new event when head rested for >2 s
Horn bud scratch	A connection of the top of head with a hind hoof. Recorded as a new event when hoof returned to ground
Postural	
Standing	A stance where all hoofs are on the ground. Recorded as duration
Lying	A position where the body is in contact with the ground. Recorded as duration
Transition	A shift from standing to lying or lying to standing
Appetitive	
Oral manipulation	An interaction between an object and the mouth, such as grooming or manipulation of fixture. Not recorded during rumination. Recorded as a new event when object left mouth for >2 s
Ruminating	A chewing jaw movement when calf was not feeding. Recorded as duration

^*a*^All behaviors are nonmutually exclusive and recorded as a frequency unless otherwise stated.

### Cortisol Analysis

Blood serum samples were shipped over night in an insulated container with frozen carbon dioxide to the Veterinary Diagnostic Laboratory (Iowa State University, Ames, IA). Samples were analyzed for cortisol (CortiCote RIA Kit, MP Biomedical, Solon, OH) in duplicate and repeated if significant differences (interassay coefficient of variation >18%) were present among duplicates. The coefficient of variation for the intra-assay variability was 17% and the coefficient of variation for the interassay variability was 13%. The limit of detection was 0.63 ng/mL.

### Statistical Analyses

All data procedures and analyses were performed using version 4.0.2 of the RStudio software (R Core Team., 2020). Pretreatment baseline values were included as covariates for analyses of behaviors and cortisol evaluated after disbudding. Baselines for each behavior represented the average of the three observations performed prior to disbudding. Four missing cortisol and 43 missing behavior (ear flicks = 10, head jerks = 7, head shakes = 7, standing = 3, transitions = 3, ruminating = 6 and oral manipulations = 7) baseline values were imputed using the sample mean within pens as described by White and Thompson (2005). Six (AD = 2, SD = 3, and TD = 1) and two (AD = 1 and TD = 1) calves were removed prior to the analyses of behaviors during and after disbudding, respectively, due to incomplete observations.

Separate models were evaluated for each outcome. All models included a covariate of *age*, a fixed effect of *treatment*, and a random intercept for *pen*. Linear mixed models for the analyses of cortisol, cauterization duration, and restraint duration were performed using the *lme* function of the *nlme* package (Pinheiro et al., 2020). Generalized linear mixed models analyzed behaviors using the *glmmTMB* function of the *glmmTMB* package (Brooks et al., 2017). For the analysis of cortisol after disbudding, the natural log transformation was applied as described by Osborne (2002).

For the analyses of outcomes evaluated after disbudding, fixed effects also included the corresponding centered and scaled *baseline* value, *time*, and *treatment × time* interaction. Only one and two calves performed horn bud scratches and head rubs at baseline, respectively; therefore, the baseline covariate was removed for these analyses. To incorporate the dependency among observations within calf, the random intercept for *calf* was added. The heterogeneous first-order autoregressive covariance structure was used for the analysis of cortisol evaluated after disbudding to account for correlated repeated measures and heteroscedasticity among times. The first-order autoregressive covariance structure was used for the analysis of behaviors evaluated after disbudding. Prior to the analyses of behaviors evaluated after disbudding, rarely observed outcomes of head shake, oral manipulation, standing, and rumination rates were aggregated into six 15-min time intervals by taking the summation of three consecutive 5-min time points. Similarly, horn bud scratch, head rub, and transition rates were seldom observed and were, therefore, summed into a single 90-min observation prior to analyses. Latency to lie down was recorded as the time lag corresponding to the first instance that lying was observed. Models for outcomes summed over all time points excluded fixed effects of *time*, *treatment × time* interaction, the random intercept for *calf*, and the covariance structure. For the analyses of behaviors evaluated during disbudding, the log of the restraint duration was an offset variable. Vocalizations, falls, and rears were observed in only 10%, 2%, and 2% of calves, respectively; and these outcomes are reported using descriptive statistics. Baseline cortisol and behaviors were analyzed separately.

For the analyses of behavior rates and latency to lie down, models were first evaluated with a Poisson distribution. Model fit was assessed by performing nonparametric overdispersion and zero-inflation tests from simulated null distributions using tools of the *DHARMa* package (Hartig, 2020); overdispersion or excess zeros were deemed present when the corresponding observation to simulation ratio was >1 (*P* < 0.05). If overdispersion was present, a negative binomial distribution with linear parameterization was used and the model was reassessed (Hardin and Hilbe, 2007). If excess zeros were present, a zero-inflated model with a single zero-inflation parameter applying to all observations was added. Poisson distributions were used for analyses of head movements and forces ahead during disbudding and ear flicks, head jerks, head rubs, head shakes, horn bud scratches, and oral manipulations after disbudding. Negative binomial distributions were used for analyses of tail wags and kicks during disbudding and transition rates and latency to lie down after disbudding. The analyses of tail wags during disbudding and horn bud scratches after disbudding included a zero-inflation factor. Beta-binomial distributions were used for analyses of standing and rumination rates after disbudding.

Maximum likelihood estimates of the model parameters were used to determine least squares means. The *F* and Wald *Χ*^2^ tests were used to test the significance of main effects for normally and nonnormally distributed outcomes, respectively. The Tukey adjustment was applied to compare groups when the corresponding main effect had *P* ≤0.05. For behavior outcomes, least squares means and confidence intervals (CIs) were transformed to the natural scale, and incidence rate ratios were used to compare groups.

## RESULTS

### Behaviors During Disbudding

Cauterization and restraint durations were consistent among treatments (Table 3). Although personnel tried to achieve the same times for cauterization and restraint between treatments, the realized time the cautery iron was in contact with the horn buds (sum of right and left horn bud) was numerically greatest for calves in the SD group. The durations of cauterization and restraint were 5.9 s [standard error (SE) = ±0.7 s] and 10.8 s (SE = ±1.3 s) when averaged across treatments, respectively.

**Table 3.** Least squares means and standard errors for effect of treatment on cauterization and restraint durations of calves undergoing disbudding procedures (*N* = 39)

	Treatment^*a*^			*F*-tests and *P*-values^*b*^	
Outcome, s	AD (*n* = 13)	SD (*n* = 12)	TD (*n* = 14)	Age (df_N_ = 1, df_D_ = 31)	Treatment (df_N_ = 2, df_D_ = 31)
Cauterization	5.6 ± 0.8	6.9 ± 0.9	5.2 ± 0.8	1.7 (0.20)	2.8 (0.07)
Restraint	11.6 ± 1.5	10.8 ± 1.5	9.9 ± 1.4	0.9 (0.35)	1.0 (0.37)

df_N_, numerator degrees of freedom; df_D_, denominator degrees of freedom.

^*a*^Treatments: AD = local anesthetic lidocaine 5 min prior to cautery disbudding; SD = sham disbudded; TD = oral tincture 2 min prior to and immediately after cautery disbudding.

Frequencies of behaviors recorded for the duration of disbudding restraint were similar among treatments (Table 4), indicating that restraint alone was a stressful event for calves and induced escape and struggle behaviors. Vocalization, fall, and rear behaviors were rarely observed. Vocalizations were not observed for calves in the AD but were observed in 7% and 23% of calves in the TD and SD groups, respectively. Falls were only observed for calves in the TD group (7%), and rears were only observed for calves in the AD group (7%).

**Table 4.** Least squares means and 95% CIs for the effect of treatment on behavior rates of calves during disbudding procedures (*N* = 39)

	Treatment^*a*^			*Χ* ^2^-tests and *P*-values	
Behavior, events per 10 s^*b*^	AD (*n* = 13)	SD (*n* = 12)	TD (*n* = 14)	Age (df = 1)	Treatment (df = 2)
Tail wags	12.5 [8.3, 18.9]	13.3 [8.9, 19.9]	13.6 [9.0, 20.5]	0.2 (0.65)	0.1 (0.95)
Head movements	2.9 [1.9, 3.9]	2.1 [1.3, 2.9]	1.9 [1.2, 2.7]	0.0 (0.97)	2.9 (0.23)
Forces ahead	0.3 [0.1, 0.8]	0.5 [0.2, 1.2]	0.5 [0.2, 1.1]	0.0 (0.89)	1.1 (0.56)
Kicks	0.5 [0.1, 1.5]	0.2 [0.0, 1.0]	0.3 [0.1, 1.1]	0.6 (0.45)	0.7 (0.69)

^*a*^Treatment: AD = local anesthetic lidocaine 5 min prior to cautery disbudding; SD = sham disbudded; TD = oral tincture 2 min prior to and immediately after cautery disbudding.

^*b*^Behavior rates are reported as the number of events per 10 s of restraint.

### Behaviors After Disbudding

Table 5 reports results for behaviors categorized into injury-directed, postural, and appetitive groups evaluated during the 360-min observation period following disbudding.

**Table 5.** Least squares means and 95% CIs for effect of treatment on behaviors of calves during the 360-min observation period following disbudding procedures (*N* = 34)

	Treatment^*a*^			*Χ* ^2^-tests and *P*-values^*b*^		
Behavior	AD (*n* = 11)	SD (*n* = 12)	TD (*n* = 11)	Tr (df = 2)	Ti (df = 17)	Tr × Ti (df = 34)
Injury directed						
Ear flicks, events per 5 min	–	–	–	4.9 (0.09)	30.7 (0.02)	72.7 (<0.01)
Head jerks, events per 5 min	2.1 [1.2, 3.5]^a^	0.9 [0.6, 1.5]^b^	1.4 [0.8, 2.4]^ab^	8.3 (0.02)	6.3 (0.99)	46.2 (0.08)
Head shakes, events per 15 min^*c*^	1.9 [1.1, 3.4]^a^	0.6 [0.4, 1.1]^b^	1.2 [0.7, 2.2]^ab^	7.7 (0.02)	2.9 (0.72)	10.3 (0.42)
Horn bud scratches, events per 90 min^*d*^	17.4 [5.9, 51.2]^a^	1.0 [0.2, 3.9]^c^	6.8 [2.2, 21.2]^b^	62.4 (<0.01)	–	–
Head rubs, events per 90 min^*d*^	1.8 [0.7, 4.6]^a^	0.6 [0.2, 1.8]^b^	2.1 [0.9, 5.2]^a^	11.5 (<0.01)	–	–
Postural						
Standing, s per 15 min^*c*^	84 [31, 205]	90 [36, 203]	62 [21, 172]	0.8 (0.67)	3.6 (0.61)	11.4 (0.33)
Transitions, events per 90 min^*c*^	4.5 [2.1, 6.9]	4.2 [2.0, 6.3]	5.3 [2.7, 8.0]	0.5 (0.78)	–	–
Latency to lie down, min	32 [25, 40]^a^	24 [19, 31]^ab^	20 [16, 26]^b^	8.0 (0.02)	–	–
Appetitive						
Ruminating, s per 15 min^*c*^	7 [1, 54]	36 [7, 165]	7 [1, 53]	2.8 (0.24)	3.0 (0.71)	13.7 (0.19)
Oral manipulations, events per 15 min^*c*^	0.4 [0.2, 0.9]	1.0 [0.5, 1.9]	0.3 [0.1, 0.8]	5.0 (0.08)	9.4 (0.09)	8.8 (0.55)

^a–c^Labeled means without a common letter within each row differ (*P* ≤ 0.05).

^*a*^Treatment: AD = local anesthetic lidocaine 5 min prior to cautery disbudding; SD = sham disbudded; TD = oral tincture 2 min prior to and immediately after cautery disbudding.

^*b*^Tr = treatment; Ti = time; Tr × Ti = treatment and time interaction.

^*c*^Observations were aggregated into six consecutive time intervals. *Χ*^2^(Ti) df = 5; *Χ*^2^(Tr × Ti) df = 10.

^*d*^Observations were aggregated over entire observational period.

#### Injury-directed Behaviors After Disbudding.

Ear flicks, head jerks, and head shakes were the most frequently observed injury-directed behaviors. In general, injury-directed behaviors were greatest for calves in the AD and lowest for calves in the SD group, while calves in the TD group had an intermediate response.

**Figure 1.**
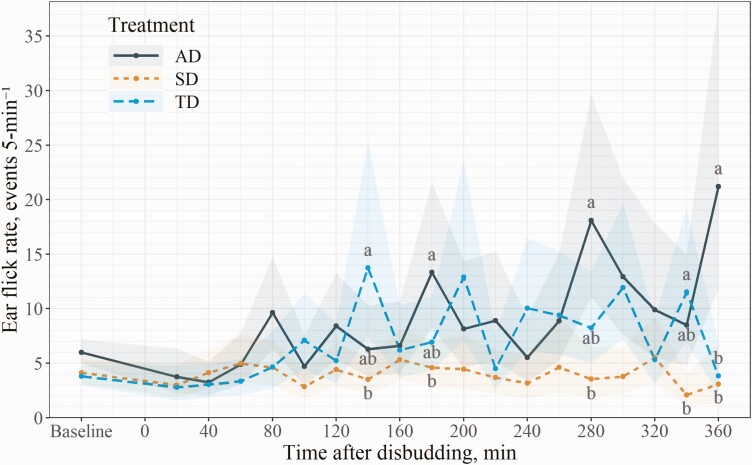
Least squares means and 80% CIs for interaction effect of treatment and time on ear flick rates of calves during the 360-min observation period following disbudding procedures (*N* = 34). The treatments were: AD = local anesthetic lidocaine 5 min prior to cautery disbudding; SD = sham disbudded; TD = oral tincture 2 min prior to and immediately after cautery disbudding. Labeled means without a common letter within each time interval differ (*P* ≤ 0.05).

There was a significant treatment and time interaction for the analysis of ear flicks, so means are reported in Figure 1. In general, the SD group had the lowest rate of ear flicks, while the AD and TD group had elevated ear flick rates following the disbudding procedure. There was an effect of baseline ear flicks (*Χ*^2^ = 6.3, *P* = 0.01) such that calves that had greater ear flicks during the pretreatment period also had greater ear flicks following the disbudding procedure. The AD group had 2.9 (95% CI = 1.0 to 8.3, *P* = 0.04), 5.1 (95% CI = 1.4 to 19.0, *P* = 0.01), and 6.9 (95% CI = 1.2 to 39.1, *P* = 0.03) times greater ear flick rates compared to the SD group at 180, 280, and 360 min after disbudding, respectively. The TD group had 3.9 (95% CI = 1.1 to 14.0, *P* = 0.03) and 5.5 (95% CI = 1.4 to 22.7, *P* = 0.01) times greater ear flick rates compared to the SD group at 140 and 340 min after disbudding, respectively. The TD and AD groups had similar (*P* ≥ 0.22) ear flick rates at all time points except at 360 min after disbudding, whereas the AD group had 5.5 (95% CI = 1.4 to 22.6, *P* = 0.01) times the ear flick rate compared to the TD group.

The AD group had a 2.3 (95% CI = 1.1 to 4.8, *P* = 0.03) times greater head jerk rate than the SD group when averaged across all time points. The TD group had comparable (*P* ≥ 0.40) head jerk rates to the other treatments throughout the experiment.

The AD group had a 3.0 (95% CI = 1.2 to 7.6, *P* = 0.01) times greater head shake rate than the SD group when averaged across all time points. The TD group had similar (*P* ≥ 0.24) head shake rates to the other groups during the experiment.

Horn bud scratches and head rubs were the least observed injury-directed behaviors, yet calves in the AD and TD groups displayed greater (*P* ≤ 0.02) frequencies compared to calves in the SD group. The AD group had the greatest horn bud scratch rate compared to the other treatments, which was 17.7 (95% CI = 6.1 to 51.4, *P* < 0.01) times greater than the SD group and 2.5 (95% CI = 1.6 to 4.2, *P* < 0.01) times greater than the TD group. Furthermore, calves in the TD scratched their horn buds at a rate that was 7.0 (95% CI = 2.2 to 21.8, *P* < 0.01) times greater than calves in the SD group. There was an effect of age on horn bud scratch rate (*Χ*^2^ = 9.4, *P* < 0.01) such that older calves were more likely to scratch their horn buds than younger calves. Head rub rates were similar (*P* = 0.86) for disbudded calves (AD and TD) regardless of treatment. The AD and TD groups had head rub rates that were 3.0 (95% CI = 1.2 to 7.8, *P* = 0.02) and 3.5 (95% CI = 1.4 to 8.7, *P* < 0.01) times greater than the SD group.

#### Postural and Appetitive Behaviors After Disbudding.

Standing and transition rates were similar among treatments, but calves in the AD took 1.6 (95% CI = 1.0 to 2.4, *P* = 0.03) times longer to lie down after the disbudding procedure compared to calves in the TD group. Oral manipulation rates and rumination rates were similar among treatments.

### Blood Serum Cortisol

**Figure 2.**
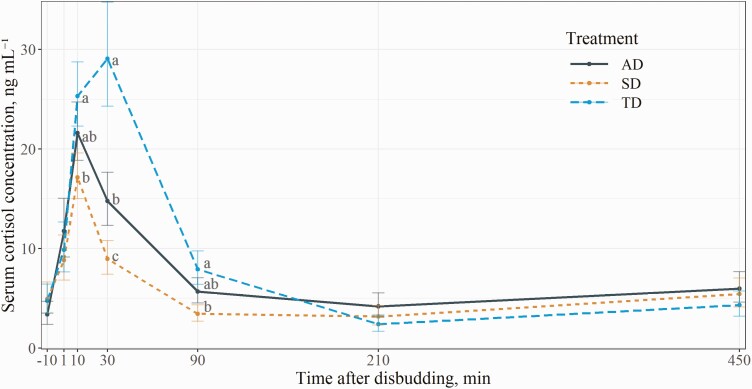
Least squares means and 80% CIs for interaction effect of treatment and sampling time on blood serum cortisol concentration (*N* = 54). The treatments were: AD = local anesthetic lidocaine 5 min prior to cautery disbudding; SD = sham disbudded; TD = oral tincture 2 min prior to and immediately after cautery disbudding. Labeled means without a common letter within each time point differ (*P* ≤ 0.05).

Blood serum cortisol concentrations were greater (*P* < 0.01) for the TD group compared to the SD group at 10, 30, and 90 min after disbudding and to the AD group at 30 min after disbudding (Figure 2). The effects of age, baseline cortisol, and the treatment × time interaction had *P* = 0.50, *P* < 0.01, and *P* < 0.01, respectively. There were no effects of age nor treatment for the analysis of baseline cortisol (*P* ≥ 0.36). The TD group had 8.2 ng/mL (95% CI = −0.4 to 16.7 ng/mL, *P* < 0.01) greater cortisol compared to the SD group 10 min after disbudding, while the AD group had an intermediate outcome. The TD group had the greatest cortisol 30 min after disbudding, which was 20.1 ng/mL (95% CI = 8.1 to 31.1 ng/mL, *P* < 0.01) and 14.3 ng/mL (95% CI = 1.5 to 27.1 ng/mL, *P* < 0.01) greater than the SD and AD groups, respectively. The AD group also had 5.8 ng/mL (95% CI = −1.1 to 12.7 ng/mL, *P* = 0.02) greater cortisol compared to the SD group at 30 min after disbudding. The TD group had 4.5 ng/mL (95% CI = 0.4 to 8.6 ng/mL, *P* < 0.01) greater cortisol compared to the SD group 90 min after disbudding, while the AD group had an intermediate response. Furthermore, the TD and AD groups had similar (*P* = 0.25) cortisol values 90 min following disbudding.

## DISCUSSION

Contrary to our hypothesis, we observed no effect of treatment on behaviors evaluated during disbudding. The relatively short cauterization duration of approximately 6 s in this experiment may explain why behavioral differences were not apparent between calves that were sham disbudded and calves that were disbudded with lidocaine but were in previous studies where the durations of cauterization were >15 s (Graf and Senn, 1999; Grøndahl-Nielsen et al., 1999). Furthermore, the level of restraint required during the disbudding procedure may have suppressed behaviors in cautery disbudded calves. Intuitively, the handler performing the disbudding procedures was not blinded to cautery versus sham disbudding. Therefore, less restraint may have been used for sham-disbudded calves, resulting in the enhanced expression of behaviors and masking of behavioral differences between cautery and sham-disbudded calves.

In general, calves disbudded with a local anesthesia had the greatest injury-directed behavioral response after disbudding, followed by calves disbudded with the tincture and sham-disbudded calves. For the calves disbudded with a local anesthetic, head jerks and head shakes peaked at approximately 80–120 min after disbudding. This time period likely represents when sensitivity in the horn bud area returned since the functional duration of lidocaine is approximately 90 min (Coetzee, 2013). Huber et al. (2013) also reported that a greater proportion of calves displayed head shakes and horn bud scratches during the 8-h observation period following disbudding when they were administered with a local anesthetic prior to disbudding compared to sham-disbudded calves.

Sham-disbudded calves had a mean ear flick rate of 3.9 events/5 min when averaged across all time points, which is greater than previous studies that report ear flick rates of ≤1.4 events/5-min (Faulkner and Weary, 2000; Stilwell et al., 2012; Huber et al., 2013). It was unclear whether these earlier studies were performed indoors where fly populations could have been suppressed. Since the current experiment took place outdoors during the summer, fly pressure and consequent avoidance behaviors may have exacerbated ear flick rates and masked differences between treatments (Eicher and Dalley, 2002). Alas, previous studies allude that ear flick behaviors may not be a completely reliable measure of pain following disbudding such that inconsistent ear flick frequency outcomes are reported among varying levels of pain mitigation therapies (Graf and Senn, 1999; Grøndahl-Nielsen et al., 1999; Faulkner and Weary, 2000; Stilwell et al., 2012; Huber et al., 2013).

Postural behavior rates of standing, lying, and transitions were similar among treatments, but calves disbudded with the tincture were more likely to lie down compared to calves disbudded with a local anesthesia. Similarly, Faulkner and Weary (2000) reported comparable lying rates among calves disbudded with varying levels of pain mitigation therapy over a 24-h observation period, and Stilwell et al. (2012) reported no effect of pain mitigation treatment on transitions between lying and standing postures. It is unclear why calves disbudded with the tincture were more likely to lie down sooner. Perhaps the first lying instance after disbudding may reflect pain in disbudded calves, but this phenomenon is currently not supported by research. The advertised calming effects of the tincture may have resulted in recumbency immediately following the procedure, which has been previously observed in disbudded calves that received a sedative (Grøndahl-Nielsen et al., 1999; Faulkner and Weary, 2000). However, plant constituents and their physiological effects have yet to be studied extensively in cattle. Potential sedation from the tincture may actually be problematic in terms of protecting animal welfare since pain-related behaviors could be concealed without actually providing any relief from pain (Stafford et al., 2003; Stilwell et al., 2010).

Appetitive behavior rates were similar among treatments. Faulkner and Weary (2000) also reported similar grooming, feeding, and drinking rates among calves disbudded with varying levels of pain mitigation therapy. An early experiment reported that cautery disbudded calves that did not receive analgesia had decreased rumination rates during the 4-h period following disbudding and increased rumination latencies compared to calves that were not disbudded (Grøndahl-Nielsen et al., 1999). Appetitive behavior differences among treatments were negligible in the current experiment and it remains unclear whether these findings were due to the level of pain or another probable cause, such as lethargy that may have decreased behavioral responses.

Calves disbudded with the experimental tincture had the greatest cortisol response, followed by calves disbudded with the local anesthesia and sham-disbudded calves. Calves that received the tincture peaked in cortisol at 30 min, whereas the calves disbudded with the local anesthesia and sham-disbudded calves peaked at 10 min after disbudding. These results are similar to those reported by Graf and Senn (1999), where cautery disbudding without analgesia resulted in a later cortisol peak compared to sham disbudding or cautery disbudding with a local anesthetic in calves. Some previous studies reported an elevated cortisol plateau for disbudded calves that received a local anesthesia (Graf and Senn, 1999; Stilwell et al., 2012; Stock et al., 2016), but this effect was not observed in the current experiment or in another similar experiment (Doherty et al., 2007). It is possible that a secondary peak in cortisol occurred but was not apparent due to straggling sample intervals.

Observed behaviors did not reflect the high cortisol levels for cautery disbudded calves that received the experimental tincture, which may have multiple plausible explanations. It is possible that unexpected inactivity and recumbency observed in calves that received the tincture could be partially explained by stress-induced analgesia and learned helplessness (Maier, 1984). Unusually low activity and inert behaviors have been previously documented in young animals following painful procedures as indicated in evaluations of chemically disbudded calves (Stilwell et al., 2008, 2009), cautery disbudded calves (Doherty et al., 2007), and castrated lambs (Molony et al., 1993).

The main possible plant-derived compound in the tincture includes a naturally occurring anti-inflammatory pro-drug (salicin) from willow tree (*S. alba*) bark (Mahdi, 2014), which is metabolized into salicylic acid in the body and has a similar anti-inflammatory mechanism to the nonsteroidal anti-inflammatories (NSAIDs) acetylsalicylic acid and sodium salicylate (Vane and Botting, 1998). Given the small quantity of tincture administered, it is unlikely that salicin had any pain-reduction effect on calves. According to Coetzee et al. (2007), a dose of 50 mg of oral acetylsalicylic acid per kilogram of body weight failed to attenuate peak cortisol concentrations after castration in 4- to 6-month-old cattle. Similarly, Mathurkar et al. (2018) reported that an oral dose of 200 mg of sodium salicylate per kilogram of body weight failed to achieve a level of salicylic acid in the blood plasma necessary to have any analgesia effect in 6-month-old sheep (*Ovis aries* L.). Another possible compound in the tincture is found in St. John’s wort (*H. perforatum*), which is commonly used as a replacement for standard anti-depressants to treat humans with mild to moderate depression (Ng et al., 2017). The main constituents presumably responsible for the anti-depressant effects of St. John’s wort are hypericin and hyperforin, yet their specific mechanisms of action are unclear and likely multifunctional (Barnes et al., 2001). Hypericin and hyperforin seem to inhibit the uptake of select neurotransmitters, such as gamma aminobutyric acid (GABA) and serotonin (Wonnemann et al., 2000). Inhibiting the uptake of GABA with gabapentin has successfully mitigated neuropathic pain in humans (Kukkar et al., 2013). Likewise, inhibiting the uptake of serotonin may mitigate acute pain as demonstrated in rodents given selective serotonin reuptake inhibitors (Singh et al., 2001; Jones et al., 2005). Few studies have investigated the analgesic effects of neurotransmitter uptake inhibitors in disbudded or dehorned calves. The combined therapy of gabapentin and the NSAID meloxicam was previously evaluated for its potential in mitigating dehorning pain in calves. While analgesic effects of the combined therapy were not outstandingly superior to other therapies, authors of these studies suggested possible synergistic pharmacokinetic properties between meloxicam and gabapentin and solicited further investigation into this phenomenon (Coetzee et al., 2011; Fraccaro et al., 2013; Glynn et al., 2013).

Regardless of the potential constituents found in the experimental tincture, numerous studies agree that systemic anti-inflammatories or opioids alone are ineffective in reducing immediate acute surgical pain on young animals as concluded under investigations with cautery disbudded calves (Caray et al., 2015), cautery disbudded goat (*Capra aegagrus hircus* L.) kids (Hempstead et al., 2020), castrated calves (Webster et al., 2013; Kleinhenz et al., 2018), and chemically disbudded calves (Stilwell et al., 2008; Braz et al., 2012; Karlen et al., 2019). Therefore, a local anesthetic should be administered to desensitize the horn bud area and effectively moderate pain during and immediately following disbudding (Grøndahl-Nielsen et al., 1999; Stilwell et al., 2008). Furthermore, when a local anesthetic is combined with a systemic NSAID, the immediate acute cortisol and injury-directed behavioral responses attenuate dramatically (Faulkner and Weary, 2000; Heinrich et al., 2009; Stilwell et al., 2012; Huber et al., 2013; Stock et al., 2016). Authors of this experiment propose that organic producers may accomplish this multimodal therapy with lidocaine as a local anesthetic and flunixin meglumine as a NSAID (Huber et al., 2013), which are both approved for use in organic livestock according to regulations set forth by the USDA-AMS-NOP (2020). Perhaps the experimental oral tincture could provide multimodal pain relief when used in combination with other validated analgesic methods, such as lidocaine; however, further evidence is required to provide any indication of its utility.

## CONCLUSIONS

Authors conclude that the restraint required for disbudding alone was a stressful event for calves, and neither the local anesthetic lidocaine nor the orally administered herbal tincture eliminated acute pain in disbudded calves as suggested by observed behaviors and blood cortisol levels. Importantly, results also suggest that additional analgesic may be required to properly manage disbudding pain effectively. The experimental tincture examined in this experiment was evidently less effective than the local anesthetic for attenuating the cortisol response following disbudding, appeared to have no mechanism to mitigate pain during the disbudding procedure, and may even suppress pain-specific behavioral responses for the hours following disbudding.
